# On-Pump FIBTEM-A5 Accurately Predicts the A5 Values After Protamine Administration

**DOI:** 10.1155/anrp/2924468

**Published:** 2025-01-29

**Authors:** Stanislaw Vander Zwaag, Matus Tomko, Tomas Madej, Jens Fassl

**Affiliations:** ^1^Department of Cardiac Anesthesiology, Heart Center Dresden, TUD Dresden University of Technology, Fetscherstrasse 76, Dresden 01307, Germany; ^2^Department of Cardiac Surgery, Heart Center Dresden, TUD Dresden University of Technology, Fetscherstrasse 76, Dresden 01307, Germany

**Keywords:** cardiac surgical procedure, fibrinogen, thromboelastometry

## Abstract

**Introduction:** In the present study, we aimed to investigate whether the measurements of the fibrinogen-dependent clot firmness in FIBTEM—one of the rotational thromboelastometry (ROTEM) assays—during cardiopulmonary bypass (CPB) accurately predict the values after protamine administration.

**Materials and Methods:** In this single-center retrospective observational study, we analyzed a database of patients undergoing on-pump surgeries between May 2022 and February 2024. We included patients in whom an intraoperative ROTEM examination during CPB and a follow-up examination directly after protamine infusion were performed. We excluded patients who received either fibrinogen concentrate or fresh frozen plasma between the two examinations. FIBTEM A5 values in both examinations were compared. The Wilcoxon signed rank test was used to compare non-normally distributed data. Linear regression analysis was used to investigate the relationship between the on-pump and postprotamine FIBTEM A5 values.

**Results:** Seventy patients were included in the statistical analysis. There was a slight but statistically significant difference between FIBTEM A5 during CPB (median 16.0 mm, IQR 10.0–22.0) and after protamine administration (median 15.5 mm, IQR 8.7–22.3, *p* = 0.021). However, in linear regression analysis, FIBTEM A5 values during the last 30 min of the extracorporeal circulation were a significant predictor of FIBTEM A5 after protamine administration (*R* = 0.902, *R*^2^ = 0.813, adjusted *R*^2^ = 0.810, F(df regression, df residual) = 295.980, *p* < 0.001). The equation: *y = *0.911*x* *+* 1 describes the line of best fit.

**Conclusions:** Within the normal range limits, there is a very strong correlation between on-pump FIBTEM A5 values and FIBTEM A5 values after protamine administration.

## 1. Introduction

Postoperative bleeding is a recognized complication of on-pump cardiac and vascular surgery. From a pathophysiological standpoint, the most significant causes of postoperative bleeding are the detrimental effects of cardiopulmonary bypass (CPB) on the coagulation cascade, anticoagulation, inflammation, fibrinolysis, and platelet dysfunction. Although heparin prevents the generation of systemic thrombin, it is unable to inhibit thrombin bound to the surface of the circuit. As a consequence, the CPB circuit surfaces develop a fibrin coating, and fibrinogen is utilized for its generation [1].

In the coagulation cascade, fibrinogen is one of the few coagulation factors that have been independently associated with postoperative chest tube output in on-pump cardiac surgery cases [2]. This finding suggests the crucial role of clot firmness in preventing postoperative blood loss. In serial examinations in on-pump coronary artery bypass graft (CABG) patients, postoperative fibrinogen levels have been shown to decline by 14% after 2 hours and by 44% after 4 hours [2, 3]. After this initial decrease, there is a rebound increase to 45% above baseline 24 h after surgery [2].

Rotational thromboelastometry (ROTEM, Werfen, Barcelona, Spain) is a viscoelastic point-of-care method for coagulation testing. In the FIBTEM assay, a reagent is added to activate the extrinsic coagulation pathway, and cytochalasin D is added to inhibit the platelet function [4]. The resulting clot firmness allows the diagnosis of hypofibrinogenemia, and it correlates with postoperative fibrinogen levels measured with the Clauss method [5–7].

Although some researchers have concluded that neither the fibrinogen level measured with the Clauss method nor FIBTEM are valid in the presence of high concentrations of heparin [8], Ji et al. have shown a strong correlation between FIBTEM A10 measurements during CPB and fibrinogen levels after separation from CPB (*R* = 0.780; *p* < 0.0001) [5].

Gronchi et al. have proven that the measurements in HEPTEM and EXTEM are valid even if heparin is present in high concentrations. However, we are not aware of any evidence supporting similar observations for FIBTEM measurements.

In the present study, we aimed to investigate whether the measurements in FIBTEM during CPB accurately predict the values after protamine administration.

## 2. Materials and Methods

Ethical approval from the Ethics Committee (Ethics Committee of the Medical Faculty of TUD Dresden University of Technology, Chairperson: Prof. B. Renner, decision number BO-EK-517122023) was obtained for this study on 1^st^ March 2024. The requirement of informed consent was waived due to local legal regulations for retrospective data analyses. All data were pseudonymised to protect patients' privacy. This study has been conducted in adherence to the Declaration of Helsinki and its amendments, as well as to the STROBE (Strengthening the Reporting of Observational studies in Epidemiology) Guidelines. The STROBE checklist is included in the Supporting Information (available here).

We retrospectively analyzed the database of patients who underwent different types of on-pump surgeries (CABG, valvular and aortic surgeries, both elective and emergent) at our single academic cardiac center in Dresden, Germany between April 2022 and February 2024. We included patients on whom an intraoperative ROTEM examination during CPB and a follow-up examination directly after protamine infusion were performed. We excluded patients who received either fibrinogen concentrate or fresh frozen plasma between the two examinations.

Cryoprecipitate is not available at our institution; therefore, it has not been administered to any patient. The decision whether to administer prothrombin complex concentrate or to perform a second ROTEM examination was made clinically on a case-to-case basis by the attending cardiac anesthesiologist.

The blood samples for on-pump testing were drawn by the perfusionists from the arterial line of CPB. The samples for the postprotamine testing were obtained from the radial arterial line. In both cases, sodium citrate blood specimen collection tubes were used for testing. We performed ROTEM examinations using semi-automatic ROTEM Sigma devices (Werfen, Barcelona, Spain).

For each patient and examination, the clot firmness values after 5 min (A5) were obtained from the device database. The statistical analysis was performed in JASP 0.18.1 (University of Amsterdam, Amsterdam, The Netherlands) [9]. We examined the normal distribution of data using the Shapiro–Wilk test, used the Wilcoxon signed rank test to analyze the differences of non-normally distributed data, and investigated the correlation between the data points with linear regression analysis. We assumed *p* values < 0.05 to be statistically significant.

## 3. Results

**Figure 1:**
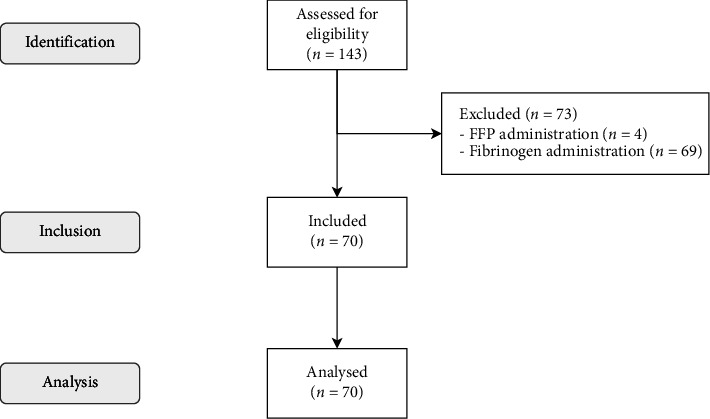
STROBE flowchart of patient selection. Abbreviations: FFP, fresh frozen plasma; STROBE, strengthening the reporting of observational studies in epidemiology.

We screened 143 patients for eligibility. We excluded 73 patients (69 due to administration of fibrinogen and 4 due to administration of fresh frozen plasma between examinations), whereas 70 patients met the inclusion criteria of the present study. There were no missing data and *n* = 70 cases were analyzed. Fourteen of the analyzed patients had an active infective endocarditis. All patients received prothrombin complex concentrate (PCC, mean dose 33 international units per kg total body weight, 95% CI [31–36]). The time elapsed between the two examinations was 37 ± 8 min. We present an overview of the inclusion process in Figure 1 and an overview of the demographic and procedural data in Table 1.

The Shapiro–Wilk test suggested a non-normal distribution of data studied, with a significant negative skew in A5-values. The median FIBTEM A5 during CPB was 16.0 mm [IQR 10.0–22.0], and 15.5 mm [IQR 8.7–22.3] after protamine administration. This difference was statistically significant in the Wilcoxon signed-rank test (*p*=0.021).

Linear regression analysis showed a statistically significant prediction of FIBTEM A5 values after protamine administration, based on A5 values during extracorporeal circulation (*R* = 0.902, *R*^2^ = 0.813, adjusted *R*^2^ = 0.810, F(df regression, df residual) = 295.980, *p* < 0.001). The equation: *y = *0.911*x *+* *1 describes the line of the best fit.

**Figure 2:**
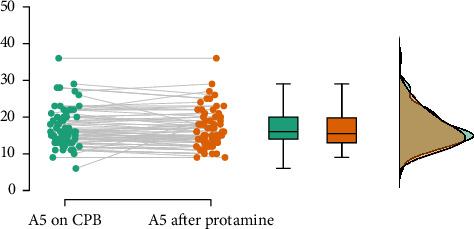
Raincloud plot of the differences between the values in both examinations.

**Figure 3:**
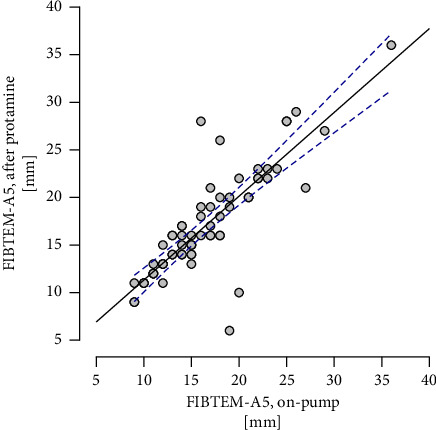
Results of FIBTEM A5 during the CPB plotted against the A5 results after protamine administration. The dashed lines indicate the 95% confidence interval.

Figure 2 presents a raincloud plot of the differences between the values in the two examinations.  Figure 3 presents the FIBTEM A5 values during the CPB plotted against the A5 after protamine administration.

## 4. Discussion

### 4.1. Main Findings and Interpretation

In this study, we demonstrated that on-pump FIBTEM measurements strongly correlate with values after protamine infusion. The *R*^2^ value of 0.813 indicates that the on-pump levels explain 81.3% of the variance of postprotamine results. The slight difference in measurements, even though statistically significant, had a magnitude of no clinical relevance.

The non-normal distribution of data seems to be caused by the retrospective nature of the study, excluding patients receiving fibrinogen between both time points. These patients were more likely to present with a low FIBTEM A5 on CPB. From the distribution plots in Figure 2, we hypothesized that including those patients would result in a normal distribution of data and, therefore, no significant differences in statistical analysis.

### 4.2. Limitations

We recognize several limitations of our work. First, due to the retrospective design of the study, the decision to re-test was made by the cardiac anesthesiologist. The indication was mostly seen in patients who had received some form of treatment after the first examination. Hence, all included patients received 4-factor prothrombin complex concentrate. To our best knowledge, however, PCC is not known to interfere with the A5 measurement in FIBTEM. Second, as the anesthesiologists provided standard care to the patients, those with FIBTEM A5 < 9 mm were more likely to receive lyophilized fibrinogen concentrate between measurements and be excluded from the study. Therefore, there are no measurements below the normal range defined by the manufacturer (i.e., FIBTEM A5 < 5 mm) in our dataset. It remains unclear whether our findings might be extrapolated to low A5 values. On the other hand, we have demonstrated that measurements within the normal range exhibit a strong correlation with values after protamine administration. Third, the measurements were made in patients undergoing different types of cardiovascular surgical procedures (i.e., CABG, valvular procedures, aortic procedures) and do not account for the possible differences in procedure types. Other factors that may influence the FIBTEM values, but have not been recorded in our study, include the remaining CPB time after the first examination, the amount of crystalloid infusion, or amount of ongoing blood loss after separation from CPB.

Finally, we did not investigate the relationship between FIBTEM A5 and clinically relevant bleeding after cardiac or major vascular surgery, nor the optimal targets to minimize postoperative blood loss. This issue will be addressed subsequently in an ongoing study at our institution.

### 4.3. Literature Review

Administration of fibrinogen concentrate has been shown to reduce the incidence of allogeneic red blood cell transfusions. No meta-analysis, however, has been able to prove that it has a significant impact on all-cause mortality [10–12]. Although the REPLACE study showed contrary results regarding the incidence of transfusions, the authors admit questionable adherence by the treating physicians to the study protocol [13]. The meta-analysis of 8 randomized control trials concluded an overall reduction of blood transfusions, but increased reoperations for bleeding in groups receiving fibrinogen concentrate [5].

Although the current evidence is insufficient to recommend prophylactic administration of fibrinogen concentrate, the post-CPB decline and increasing drainage output with low fibrinogen levels warrant further research to identify the optimal fibrinogen levels in patients recovering from on-pump surgery. Viscoelastic diagnostic methods such as ROTEM allow clinicians to quickly assess the fibrinogen-dependent test and supplement blood-derived products in a prompt manner.

ROTEM is a point-of-care diagnostic test. However, it may take up to 15 min to obtain A5 results, and another 15 min to obtain lyophilized fibrinogen concentrate and dilute it at the bedside. In a busy cardiac operating room after separation from CPB, this labor-intensive preparation may redirect the attention of the anesthetic team away from the patient. In selected cases, knowing that the on-pump FIBTEM measurements are comparable to those after protamine administration may allow clinicians to prepare the infusion while still on CPB, and therefore optimize the management after separation from CPB.

Even though most studies utilize FIBTEM A10 to guide substitution, a few researchers have demonstrated an excellent correlation between A5 and A10 values, both in trauma and cardiac patients [14, 15]. Due to logistical reasons, as a high-volume center with one ROTEM-device, we discontinue the majority of our tests after obtaining the A5 results in all four modalities, and base our clinical decisions on those values.

Recently, research has focused on the exact trigger and target values for fibrinogen substitution. Monaco et al. studied patients undergoing open thoraco-abdominal aortic aneurysm repairs. They concluded that A10 less than 3 mm was an independent risk factor for extensive bleeding and identified a cut-off value of 9 mm upon analysis of the receiver operator characteristics curve [16]. In other studies, cut-offs of 8 mm [17] and 10 mm [18] also were used to guide supplementation.

### 4.4. Future Perspectives

In the present research article we have shown the usefulness of on-pump FIBTEM A5 to guide supplementation therapy after protamine administration. However, it remains unclear if the patients benefit from ROTEM-based administration of coagulation factors before the diagnosis of sustained microvascular bleeding after protamine could be made. Moreover, the exact target FIBTEM A5 levels, their relationship to clinical outcomes and the dose of fibrinogen concentrate necessary to achieve them are yet to be determined.

## 5. Conclusions

Within the normal range limits, there is a very strong correlation between on-pump FIBTEM A5 values and FIBTEM A5 values after protamine administration. The values obtained during extracorporeal circulation can be utilized to guide fibrinogen administration after weaning from CPB. Further research is needed to evaluate the correlation below the normal range limits, the clinical relevance of the measured values, and the optimal levels of FIBTEM A5 in surgical patients.

## Figures and Tables

**Table 1:** Overview of demographic and procedural data.

**Gender**	***n* (%)**

Female	12 (17%)
Male	58 (83%)

**Demographic data**	**Mean ± SD**

Age	67 ± 12 years
Height	175 ± 8 cm
Weight	86 ± 20 kg

**Procedural data**	***n* (%)**

Type of surgery	
CABG	8 (11%)
Single valve	22 (31%)
Single valve + CABG	9 (13%)
Single valve + aorta	8 (11%)
Single valve + aorta + CABG	1 (1%)
Two valves	7 (10%)
Two valves + CABG	3 (4%)
Three valves	1 (1%)
Aorta	8 (11%)
LVAD-implantation	1 (1%)
TAH-implantation	1 (1%)
Re-exploration for tamponade	1 (1%)
Re-do surgery	14 (20%)
Urgency	
Elective	35 (50%)
Urgent	25 (36%)
Emergent	10 (14%)
Active endocarditis	14 (20%)

Abbreviations: CABG, coronary artery bypass graft; LVAD, left ventricular assist device; TAH, total artificial heart.

## Data Availability

All studied data might be obtained from the corresponding author upon a reasonable request.

## Supporting Information

The STROBE (Strengthening the Reporting of Observational studies in Epidemiology) checklist is included in the Supporting Information.

## References

[1] Paparella D., Brister S. J., Buchanan M. R. (2004). Coagulation Disorders of Cardiopulmonary Bypass: A Review. *Intensive Care Medicine*.

[2] Ternström L., Radulovic V., Karlsson M. (2010). Plasma Activity of Individual Coagulation Factors, Hemodilution and Blood Loss After Cardiac Surgery: A Prospective Observational Study. *Thrombosis Research*.

[3] Momeni M., Carlier C., Baele P. (2013). Fibrinogen Concentration Significantly Decreases After On-Pump Versus Off-Pump Coronary Artery Bypass Surgery: A Systematic Point-of-Care ROTEM Analysis. *Journal of Cardiothoracic and Vascular Anesthesia*.

[4] Lang T., Toller W., Gütl M. (2004). Different Effects of Abciximab and Cytochalasin D on Clot Strength in Thrombelastography. *Journal of Thrombosis and Haemostasis*.

[5] Ji S.-M., Kim S.-H., Nam J.-S. (2015). Predictive Value of Rotational Thromboelastometry during Cardiopulmonary Bypass for Thrombocytopenia and Hypofibrinogenemia After Weaning of Cardiopulmonary Bypass. *Korean Journal of Anesthesiology*.

[6] Loesche W., Reinhöfer M., Macholdt C. (2007). ROTEM® Thrombelastometry in On-Pump Cardiac Surgery Patients. *Critical Care*.

[7] Gauger M. S., Kaufmann P., Kamber F. (2022). Rotational Thromboelastometry Values After On-Pump Cardiac Surgery-A Retrospective Cohort Study. *Seminars in Cardiothoracic and Vascular Anesthesia*.

[8] Gertler R., Wiesner G., Tassani-Prell P., Braun S.-L., Martin K. (2011). Are the Point-of-Care Diagnostics MULTIPLATE and ROTEM Valid in the Setting of High Concentrations of Heparin and its Reversal With Protamine?. *Journal of Cardiothoracic and Vascular Anesthesia*.

[9] (2024). JASP Team. JASP. https://jasp-stats.org/.

[10] Rahe-Meyer N., Pichlmaier M., Haverich A. (2009). Bleeding Management With Fibrinogen Concentrate Targeting a High-Normal Plasma Fibrinogen Level: A Pilot Study. *British Journal of Anaesthesia*.

[11] Rahe-Meyer N., Solomon C., Winterhalter M. (2009). Thromboelastometry-Guided Administration of Fibrinogen Concentrate for the Treatment of Excessive Intraoperative Bleeding in Thoracoabdominal Aortic Aneurysm Surgery. *The Journal of Thoracic and Cardiovascular Surgery*.

[12] Rahe-Meyer N., Solomon C., Hanke A. (2013). Effects of Fibrinogen Concentrate as First-Line Therapy During Major Aortic Replacement Surgery: A Randomized, Placebo-Controlled Trial. *Anesthesiology*.

[13] Rahe-Meyer N., Levy J. H., Mazer C. D. (2016). Randomized Evaluation of Fibrinogen vs Placebo in Complex Cardiovascular Surgery (REPLACE): A Double-Blind Phase III Study of Haemostatic Therapy. *British Journal of Anaesthesia*.

[14] Blayney A., McCullough J., Wake E. (2022). Substitution of ROTEM FIBTEM A5 for A10 in Trauma: An Observational Study Building a Case for More Rapid Analysis of Coagulopathy. *European Journal of Trauma and Emergency Surgery*.

[15] Olde Engberink R. H. G., Kuiper G. J. A. J. M., Wetzels R. J. H. (2014). Rapid and Correct Prediction of Thrombocytopenia and Hypofibrinogenemia With Rotational Thromboelastometry in Cardiac Surgery. *Journal of Cardiothoracic and Vascular Anesthesia*.

[16] Monaco F., Barucco G., Licheri M. (2021). Trigger and Target for Fibrinogen Supplementation Using Thromboelastometry (ROTEM) in Patients Undergoing Open Thoraco-Abdominal Aortic Aneurysm Repair. *European Journal of Vascular and Endovascular Surgery*.

[17] Monaco F., Barucco G., Nardelli P. (2019). Editor’s Choice-A Rotational Thromboelastometry Driven Transfusion Strategy Reduces Allogenic Blood Transfusion During Open Thoraco-Abdominal Aortic Aneurysm Repair: A Propensity Score Matched Study. *European Journal of Vascular and Endovascular Surgery*.

[18] Monaco F., Nardelli P., Denaro G. (2020). First Experience with a ROTEM-Enhanced Transfusion Algorithm in Patients Undergoing Aortic Arch Replacement With Frozen Elephant Trunk Technique. A Theranostic Approach to Patient Blood Management. *Journal of Clinical Anesthesia*.

